# The ‘Lifeboat Hypothesis’: Aquatic Microplastics in a Warming World—Climate‐Resilient Refugia for Bacterial Pathogens

**DOI:** 10.1111/gcb.71078

**Published:** 2026-08-25

**Authors:** Richard S. Quilliam, Michael J. Ormsby

**Affiliations:** ^1^ Biological and Environmental Sciences University of Stirling Stirling UK; ^2^ Biodiversity, One Health and Veterinary Medicine University of Glasgow Glasgow UK

**Keywords:** antimicrobial resistance, climate change, one health, pathogen dispersal, planetary health, plastisphere

## Abstract

The *Lifeboat hypothesis* proposes that microplastics act as mobile microbial refugia, buffering environmental stress and enabling persistence, adaptation and dispersal of microorganisms, including pathogens and antimicrobial resistance (AMR) determinants. Microplastic‐associated biofilms (the plastisphere) mitigate UV radiation, osmotic stress and environmental fluctuations, while promoting stress responses and horizontal gene transfer. Ocean circulation then facilitates long‐range transport of these communities, linking distant ecosystems. Climate‐driven cryosphere thaw may further introduce ancient microorganisms into the contemporary plastisphere (‘paleo‐plastisphere’), where they are captured and redistributed. In parallel, ingestion by marine organisms provides a biological bypass that enhances microbial survival and accelerates trophic transfer. Collectively, these processes position microplastics as dynamic vectors of microbial connectivity, with implications for infectious disease exposure, biosecurity leakage and transboundary AMR dissemination under global environmental change.

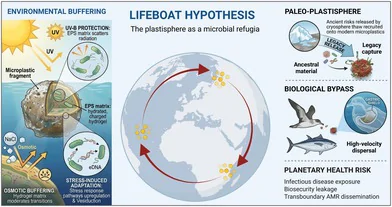

## 1 Introduction

The stability of Earth's natural systems is being undermined by the global redistribution of synthetic plastic polymers. Microplastics (< 5 mm) are no longer merely environmental contaminants; they are ubiquitous structural components of the global hydrosphere (Geyer et al. 2017; Horton and Dixon 2018), with their distribution so extensive that they are now considered a primary stratigraphic indicator of the current geological epoch (Zalasiewicz et al. 2016).

Importantly, the recalcitrant nature of plastics allows diverse, complex, multi‐generational microbial biofilms to develop on their surfaces (Ormsby and Quilliam 2026). While much attention has focused on the taxonomic diversity of plastic‐associated microbial communities (the ‘plastisphere’) compared to natural substrates (Amaral‐Zettler et al. 2020; Guruge et al. 2024; Li et al. 2024); their enzymatic potential to degrade synthetic polymers (Chamas et al. 2020; Miao et al. 2023); and the toxicological impacts of plastic ingestion (Abahussain et al. 2025), a more subtle threat remains as a critical biosecurity blind spot: the role of the plastisphere as a mobile, climate‐resilient reservoir for human and animal pathogens remains.

Climate change provides the environmental context in which the Lifeboat hypothesis becomes increasingly relevant. Intensified flooding, altered river discharge, changing ocean circulation and sea‐ice loss are reshaping both the distribution of aquatic pathogens and the transport pathways of floating plastics. Rather than acting independently, climate change and plastic pollution may therefore interact to increase opportunities for long‐distance pathogen dispersal.

Historically, the long‐distance dispersal of aquatic pathogens was constrained by geo‐spatial environmental filters including temperature gradients, ultraviolet radiation, nutrient limitation and salinity transitions, which collectively reduced survival during their transfer. Traditionally, ‘rafting’ on floating debris has been recognised as a primary mechanism for the stochastic introduction of invasive macro‐biota, such as sea squirts, sponges and bivalves, into novel habitats. While these processes have historically reshaped coastal biodiversity, they are typically viewed as slow and erratic. However, climate change is reshaping these constraints during their transfer (Martiny et al. 2006; Sosah et al. 2025), with rising sea surface temperatures and more extreme weather events not only expanding the habitable range of pathogens like *Vibrio* spp. (Baker‐Austin et al. 2024; Brumfield et al. 2025), but also accelerating the mobilisation of plastics from urban centres into the marine environment (Cheung and Not 2023; Kelly et al. 2025).

**FIGURE 1:**
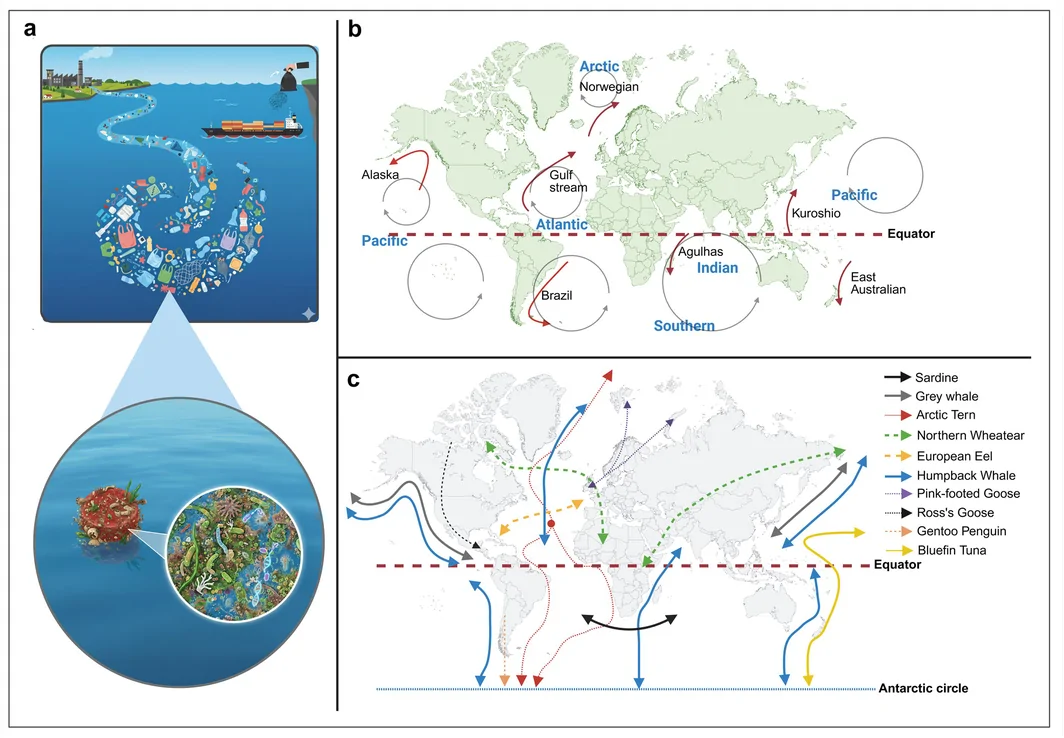
Microplastics as a planetary‐scale dispersal infrastructure. (a) Conceptual schematic of the ‘Lifeboat’ hypothesis: Microplastics acquire microbial biofilms (the plastisphere) that may extend bacterial survival during long‐distance transport. (b) Major ocean currents and subtropical gyres that facilitate movement of plastic‐associated microbial communities. (c) Example migratory pathways of fish, seabirds and marine mammals intersecting high‐density plastic zones, potentially enabling ingestion‐mediated microbial transport.

Here, we propose the ‘Lifeboat hypothesis’, where microplastics act as a synthetic survival mechanism that may permit subsets of pathogens to endure environmental pressures that would otherwise act as natural biocides. This hypothesis is intended to synthesise existing mechanisms into a unified, testable framework, rather than to propose a singular novel process. In this framework, plastic‐associated biofilms may buffer environmental stressors that would otherwise reduce microbial viability during dispersal and allow subsets of transported bacteria to remain viable across longer distances and broader environmental gradients (Figure 1a). In ecological terms, successful dispersal requires microorganisms to survive environmental stress while retaining sufficient physiological capacity for subsequent colonisation. Attachment to microplastic biofilms may reduce this dispersal‐associated fitness cost by buffering mortality drivers including ultraviolet irradiation, salinity transitions and nutrient limitation. Rather than simply increasing survival, the Lifeboat hypothesis proposes that plastisphere communities preserve the physiological competence required for successful establishment after arrival.

Emerging evidence suggests that attachment to plastic‐associated biofilms may provide a mechanistic basis for reduced dispersal‐associated fitness costs. Extracellular polymeric substance (EPS) matrices are known to buffer microorganisms from environmental stress through the creation of hydrated microenvironments and diffusion barriers (Flemming and Wingender 2010). Consistent with this principle, plastisphere communities differ functionally from free‐living assemblages and have been proposed as substrates that facilitate the persistence and long‐distance transport of pathogenic microorganisms (Bowley et al. 2021; Li et al. 2024). Experimental observations further indicate that plastic‐associated pathogens can exhibit enhanced survival under ultraviolet stress and during transfer across environmental gradients (Metcalf et al. 2023; Ormsby et al. 2024), supporting the hypothesis that plastisphere attachment may preserve physiological competence during dispersal.

Importantly, we are not proposing that plastics are acting as primary drivers of disease emergence, and we explicitly caution against interpreting association as causation in the absence of controlled experimental validation. Instead, we propose that plastics act as exposure modifiers that may extend survival windows for transported pathogenic bacteria. We emphasise that the strength and generality of the Lifeboat effect will likely vary across environmental contexts, polymer types and microbial taxa. By reframing microplastics as exposure modifiers rather than passive debris, we provide a necessary lens for public health surveillance and planetary governance in our increasingly plasticised world.

## 2 Microplastics as Persistent Microbial Substrates

Within the Lifeboat framework, microplastics are not passive rafts; they are stable microbial refugia. By providing a stable, high‐surface‐area substrate, synthetic polymers fundamentally alter the physiological state and survival probability of the microbes they carry. Here, we synthesise four critical mechanisms that may contribute to microplastics functioning as microbial transport vectors:

### 2.1 Biofilm Formation, EPS Protection and UV Tolerance

In surface waters, UV‐B radiation acts as a primary natural biocide, rapidly neutralising planktonic bacteria. Within the plastisphere, however, the biofilm EPS matrix functions as a photoprotective barrier. By adsorbing dissolved organic matter and scattering radiation, this matrix may permit pathogens, including pathogenic *Vibrio* spp. and enteric *Salmonella* spp., to maintain viability in high‐energy surface currents (Bowley et al. 2021; Ormsby et al. 2024). Crucially, this protection is linked to a substrate‐induced phenotypic shift: attachment to plastic has been associated with the upregulation of DNA repair and stress‐response pathways; metabolic investments that are typically energetically unfeasible for free‐floating cells (Arias‐Andres et al. 2018; Ogonowski et al. 2018; Tao et al. 2024). However, a balanced evaluation of these mechanisms must account for potential antagonistic pressures. While biofilms offer physical shielding, the metabolic costs of maintaining complex community structures in nutrient‐poor open oceans may limit long‐term viability. Additionally, the presence of antimicrobial additives or leachates within certain plastic polymers could potentially inhibit microbial growth, suggesting that the ‘Lifeboat’ effect may be highly context‐dependent.

### 2.2 Osmotic Buffering in Transitional Waters

The freshwater–marine continuum represents a significant physiological barrier, where sudden salinity shifts typically trigger rapid cell lysis in terrestrial and enteric taxa. Plastisphere biofilms function as interstitial osmotic buffers, with the hydrated EPS matrix acting as a charged hydrogel, moderating ion exchange rates and slowing the diffusion of solutes (Flemming and Wingender 2010). Bacteria on microplastics can respond to these transitions by over‐secreting EPS, which provides the necessary temporal window for cellular acclimation and prevents osmotic shock (Amaral‐Zettler et al. 2015; Metcalf et al. 2023).

### 2.3 Horizontal Gene Transfer (HGT) and Stress‐Induced Adaptation

The high‐density microbial architecture of the biofilm, combined with the capacity of plastic to adsorb co‐contaminants such as pharmaceuticals, heavy metals and persistent organic pollutants, may create environments of elevated selective pressure, that could facilitate HGT (Larsson and Flach 2022; Shin et al. 2023). Conjugation rates for clinically relevant plasmids (e.g., *NDM‐1*) are up to 200 times higher in the plastisphere than in the surrounding water column (Yang et al. 2025). Furthermore, hydrophobic polymers like PET can stabilise eDNA, extending the ‘genetic half‐life’ of antimicrobial resistance genes (ARGs) and facilitating their transfer via stress‐induced outer membrane vesicles, a process known as vesiduction (Sharma et al. 2024). In this context, microplastics acting as Lifeboats may not only transport microbial communities but could also influence their genotypic adaptation under certain conditions.

### 2.4 Acid Tolerance and the Gastrointestinal Biological Bypass

Microplastics may act as protective conduits during ingestion by marine and migratory fauna (Figure 1c). When consumed by marine mammals, birds, or fish, the complex micro‐topography and biofilm structure could shield embedded bacteria from the extreme acidity and enzymatic degradation of the gastrointestinal tract (Cheng et al. 2023; Jawdhari et al. 2024). This ‘biological bypass’ permits the survival of pathogens through the host's primary digestive defences. Once excreted into nutrient‐rich faecal plumes, these primed pathogens could be pre‐positioned for downstream colonisation in geographically distant ecosystems.

However, it is critical to note that the benefits conferred by this ‘Lifeboat’ are not monolithic. The degree of environmental buffering is likely contingent on specific microbial physiological traits; for instance, Gram‐positive bacteria with robust cell walls may derive different fitness advantages from biofilm shielding than more fragile Gram‐negative pathogens. Future research must delineate these taxa‐specific survival strategies to avoid overgeneralising the plastisphere's role as a universal vector.

## 3 Climate Change as an Accelerator of the Lifeboat Effect

Anthropogenic climate change is systematically modifying the environmental filters that historically governed microbial biogeography. We identify two primary climate‐driven accelerators that could amplify the impacts of the microplastic Lifeboat: the emergence of the Paleo‐Plastisphere and the restructuring of oceanic dispersal corridors. We hypothesise the emergence of a ‘Paleo‐Plastisphere’ interface, where modern plastics act as vectors for the capture and redistribution of legacy biological material. As the cryosphere thaws, plastics may facilitate the transport of ancestral pathogens or antimicrobial resistance genes (ARGs) into modern ecosystems. While direct empirical evidence for this interface is currently emergent, it represents a high‐priority, testable proposition for evaluating how plastic pollution mediates the ‘resurrection’ of dormant microbial risks. At present, this concept remains speculative and is intended to highlight a potential research direction rather than a demonstrated environmental process.

The accelerated thawing of the cryosphere, including permafrost and glacial ice, is discharging a significant biological legacy into modern hydrological networks. This includes viable ancient microorganisms, spores and preserved genetic material, such as ancestral ARGs (Caruso and Rizzo 2025; D'Costa et al. 2011; Yarzábal et al. 2021). Metagenomic catalogues of glacier‐fed systems have already identified nearly 1000 novel bacterial species being released into the global water cycle, many of which harbour these legacy traits (Liu et al. 2022). When this legacy biological material encounters contemporary microplastics, the resulting Paleo‐Plastisphere could create a temporal bridge, facilitating the integration of ancient genetic sequences into modern microbial plastisphere communities. Microplastic biofilms provide the structural stability required to facilitate the integration of ancient genetic sequences into modern microbial communities via HGT. By capturing and recruiting these dormant biological agents, synthetic polymers may permit the re‐emergence of ancestral traits within contemporary ecosystems: a process that could potentially influence the evolutionary trajectory of human and animal pathogens.

Simultaneously, oceanic warming and altered circulation patterns are recalibrating the viability windows for transported pathogens. For example, increases in sea surface temperature may extend pathogen‐specific survival half‐lives, thereby increasing the probability of successful transport over a given current velocity and distance. The intensification of western boundary currents and the poleward shift of subtropical gyres act as high‐speed conveyor belts, reducing the duration of exposure to harsher pelagic conditions (van Sebille et al. 2020; Yang et al. 2016) (Figure 1b). The empirical reality of these long‐distance dispersal routes is highlighted by the recovery of decades‐old ‘legacy’ plastics; such as 60‐year‐old Canadian household containers washing up on the Scottish coastline, illustrating how synthetic architecture can persist across trans‐Atlantic scales (BBC 2026). Furthermore, as sea surface temperatures rise, the thermal gradient between low and high latitudes is eroding, which was once a formidable barrier for both tropical and temperate pathogens. Such thermal homogenisation, combined with the protective buffering of the plastisphere, allows for the poleward range expansion of taxa such as *Vibrio* spp. into regions that were previously ecologically inaccessible (Froelich and Daines 2020; Vezzulli et al. 2013). In this warming world, the ‘Lifeboat’ does not just travel further; the waters it traverses are becoming increasingly hospitable to its cargo.

The role of climate change as a mechanistic accelerator may be further influenced by increased thermocline stratification. Warming surface waters can keep plastic particles and their associated pathogenic payloads within the photic zone for longer durations. This maximises the window for both biofilm maturation and high‐velocity transport via surface currents, potentially lowering the environmental barriers to colonisation under favourable conditions.

## 4 Geopolitical Vulnerability and the Governance of the Microbial Commons

The Lifeboat hypothesis exposes a critical blind spot in global biosecurity. From a risk‐management perspective, the Lifeboat hypothesis suggests that microplastics should be integrated into proactive biosecurity surveillance. Rather than viewing plastic solely as a waste management issue, it should be considered a non‐traditional factor in infectious disease modelling, particularly in regions where aquaculture and human interfaces intersect with high plastic flux. By facilitating geopolitical bypasses, microplastics permit pathogens and ancient genetic material to traverse international borders with impunity, potentially limiting the effectiveness of localised environmental management. This creates a paradigm of shared vulnerability: even nations with exemplary environmental standards remain susceptible to an exposure pulse of microbes from distant sources via transboundary ocean currents or migratory fauna. This poses a direct threat to critical high‐latitude receptors, specifically salmon aquaculture industries and coastal cities in Northern Europe and North America. As warming waters erode historical thermal barriers, these regions face a tangible risk of ‘biosecurity leakage’ where resilient, plastic‐borne pathogens undermine local food security and public health standards. Addressing this synthetic‐biological threat requires a radical shift towards the transboundary coordination of the microbial commons.

Governance must move beyond our current focus on macro‐pollution; the forthcoming Global Plastic Treaty may benefit from incorporating plastisphere monitoring approaches, enshrining microbial metrics and pathogen‐loading capacities within international law. Furthermore, cryospheric protection could be considered within a broader biosecurity framework to prevent the re‐emergence of ancestral traits within contemporary ecosystems. Integrating these dynamics into One Health frameworks may become increasingly important for robust risk assessment in a world where the boundaries of microbial life are being redrawn by the pervasiveness of plastic pollution.

## 5 Conclusions and a Research Roadmap

The Lifeboat hypothesis redefines microplastics from a static debris problem into a dynamic, climate‐accelerated biological threat. While substantial empirical uncertainty remains regarding effect size, frequency and dominant pathways, the convergence of plastic persistence, microbial ecology and climate‐driven redistribution warrants proactive integration into planetary health surveillance and governance frameworks. As we have argued, the synergy between plastic persistence and climate‐driven range shifts may create a novel vector for pathogens that bypasses historical ecological barriers. This transition from pollutant to vector requires a paradigm shift in how we monitor and manage aquatic health. We propose a priority research roadmap:

### 5.1 Integrated Bio‐Transport Modelling Across Environmental and Epidemiological Domains

Current modelling frameworks treat plastic transport and infectious disease dynamics as largely independent problems. Oceanographic and hydrological models can predict the trajectories, residence times and accumulation zones of plastic particles, while epidemiological models focus on host–pathogen interactions and transmission within human or animal populations. The Lifeboat Hypothesis highlights the need to bridge these approaches.

We propose the development of integrated bio‐transport models that incorporate biological persistence alongside physical movement. Central to this effort would be the estimation of Plastisphere Survival Coefficients (PSCs): empirically derived parameters describing the extent to which association with plastic‐associated biofilms extends the viable half‐life of specific microbial taxa under defined environmental stressors (e.g., UV exposure, salinity fluctuation, nutrient limitation). The PSC is defined as the ratio of the decay rate of a pathogen within a plastisphere biofilm relative to its decay rate in a free‐living state under identical environmental stressors. A Lifeboat effect is validated where PSC < 1, signifying enhanced persistence. This metric allows for the rigorous comparison of ‘refugia efficiency’ across different polymer types and environmental gradients. This formulation provides a falsifiable criterion: if no consistent difference in decay rates is observed between plastic‐associated and free‐living states across controlled conditions, the Lifeboat hypothesis would not be supported. Incorporating PSCs into particle‐tracking models would allow estimation of not just where plastics travel, but whether they are likely to arrive carrying viable microbial cargo. These frameworks should also include asynchronous arrival variables, capturing scenarios in which pathogens reach new regions before local environmental conditions independently permit their persistence. Such temporal mismatches are particularly relevant for rapidly warming high‐latitude and high‐altitude systems, where surveillance and clinical awareness may lag behind ecological change. To quantify this, future research must establish a standardised pathogen loading capacity for different polymer types (e.g., polyethylene vs. polystyrene). This metric should define the density of specific pathogens a plastic surface can support across a range of UV and salinity stressors, providing the empirical foundation for the ‘Lifeboat Survival Coefficients’ needed in predictive biosecurity models.

Future models must also account for the projected tripling of plastic production by 2060, which will significantly increase the density of this dispersal infrastructure. Instead of broad taxonomic surveys, research must prioritise the functional plasticity of the Plastisphere. We need to quantify how specific polymer architectures (e.g., highly weathered vs. virgin pellets) influence the metabolic ‘hibernation’ or ‘activation’ of pathogens during long‐range transport. This shift from ‘who is there’ to ‘what are they capable of upon arrival’ is the missing link in biosecurity modelling.

### 5.2 The Gastric Shield and Ingestion‐Mediated Transport

A second priority is to resolve the mechanisms by which plastic‐associated microorganisms move between abiotic vectors and living hosts. While ingestion of microplastics by wildlife is well documented, the biological consequences of this process for pathogen persistence remain poorly understood. Research should prioritise ingestion‐mediated transport, examining how gastrointestinal environments of migratory birds, marine mammals and fish influence the viability, metabolic state and genetic composition of plastic‐associated biofilms. Key questions include whether gut passage selects for stress‐tolerant or virulent phenotypes, whether HGT is promoted or suppressed and how long viable microorganisms persist post‐egestion.

These studies should be spatially targeted to interfaces where migratory flyways intersect with high‐density plastic accumulation zones, such as major estuaries, frontal systems and subtropical gyres. Linking movement ecology with microbiological assays would allow identification of biological ‘short‐circuit’ pathways that bypass slower physical transport routes, with direct implications for zoonotic and food‐borne risk. We must prioritise in vitro digestion models that simulate the specific gastric conditions of migratory vectors (e.g., the high‐acidity gizzards of seabirds). Research should quantify the ratio of viable pathogens entering vs. exiting the GI tract when associated with different polymer types. Establishing survival ratios will allow us to move from anecdotal observations of ingestion to predictive models of ‘biosecurity leakage’ caused by migratory wildlife.

### 5.3 In Situ and Mesocosm Experiments Under Realistic Multi‐Stressor Regimes

Most experimental studies of the plastisphere rely on static laboratory conditions that poorly represent the dynamic and stressful environments encountered during long‐distance transport. The Lifeboat hypothesis explicitly depends on the interaction of multiple stressors acting simultaneously. We therefore advocate for in situ and mesocosm experiments that expose plastic‐associated biofilms to the combined ‘triple threat’ characteristic of open‐water transport: fluctuating ultraviolet radiation, rapid salinity transitions and sustained nutrient limitation. Such systems would enable real‐time observation of physiological adaptation, including activation of stress responses, maintenance of membrane potential and rates of HGT under climate‐relevant conditions.

Importantly, these experiments should prioritise functional outcomes, such as retention of infectivity, resistance profiles, or colonisation capacity, rather than taxonomic shifts alone. This focus aligns experimental outputs with One Health endpoints and avoids over‐interpretation of compositional data. These experiments should also quantify the balance between climate‐driven increases in plastic weathering and climate‐driven transport efficiency. Although polymer ageing may alter microbial attachment and persistence, degradation occurs over different temporal scales than environmental transport. Determining how these opposing processes interact will define an important boundary condition of the Lifeboat hypothesis.

### 5.4 Auditing Legacy Interfaces and the Modern Paleo‐Plastisphere

Finally, targeted surveillance is required at interfaces where thaw‐released biological material encounters modern plastic pollution. While plastics themselves are not ancient, cryosphere retreat is mobilising microorganisms and genetic elements that have been environmentally sequestered for extended periods. Modern plastics may act as contemporary interfaces that enable their reintegration into active microbial networks.

We propose metagenomic and viability‐focused audits of plastics collected from meltwater‐influenced rivers, proglacial lakes, tundra runoff and downstream coastal systems. These efforts should aim to identify whether plastic‐associated biofilms are enriched for thaw‐derived antibiotic resistance genes, stress‐tolerant taxa, or spore‐forming organisms. Attention should be paid to potential ‘double‐armoured’ threats, where inherent microbial resilience (e.g., spores or extremotolerant phenotypes) is further enhanced by plastisphere‐mediated protection. Identifying such combinations would allow prioritisation of monitoring efforts and inform risk‐based governance approaches.

Taken together, these research priorities shift the focus from descriptive cataloguing of plastic‐associated microbes toward quantitative, mechanism‐driven assessment of risk. By embedding biological persistence within transport models, resolving biotic–abiotic transfer pathways and aligning experiments with realistic environmental stressors, the Lifeboat hypothesis can be tested, refined and translated into actionable insights for planetary health governance.

## Author Contributions

**Richard S. Quilliam:** writing – review and editing, conceptualization. **Michael J. Ormsby:** conceptualization, writing – original draft, visualization, writing – review and editing, project administration.

## Conflicts of Interest

The authors declare no conflicts of interest.

## Data Availability

Data sharing not applicable to this article as no datasets were generated or analysed during the current study.
